# Efficacy and Safety of Acupuncture-Moxibustion Therapy on Chemotherapy-Induced Leukopenia: A Systematic Review and Meta-Analysis

**DOI:** 10.1155/2020/5691468

**Published:** 2020-10-30

**Authors:** Huimin Jin, Yuqian Feng, Yuying Xiang, Yiting Zhang, Wurong Du, Harpreet S. Wasan, Shanming Ruan, Dawei Huang

**Affiliations:** ^1^First Clinical Medical College of Zhejiang Chinese Medical University, Hangzhou 310051, Zhejiang, China; ^2^Department of Cancer Medicine, Hammersmith Hospital, Imperial College Healthcare NHS Trust, London, UK; ^3^Department of Medical Oncology, The First Affiliated Hospital of Zhejiang Chinese Medical University, Hangzhou 310006, Zhejiang, China; ^4^Department of Chinese Medicine, The First Affiliated Hospital of Zhejiang Chinese Medical University, Hangzhou 310006, Zhejiang, China

## Abstract

**Background:**

Acupuncture-moxibustion therapy (AMT), as an integral part of complementary and alternative medicine, has been used for centuries in treatment of numerous diseases. Nevertheless, there is no available supportive evidence on the efficacy and safety of acupuncture-moxibustion therapy in patients with chemotherapy-induced leukopenia (CIL). The purpose of this study is to evaluate the efficacy and safety of acupuncture-moxibustion therapy in treating chemotherapy-induced leukopenia.

**Methods:**

Relevant studies were searched in nine databases up to September 19, 2020. Two reviewers independently screened the studies for eligibility, extracted data, and assessed the methodological quality of selected studies. Meta-analysis of the pooled mean difference (MD) and risk ratio (RR) with their respective 95% confidence intervals (CI) were calculated.

**Results:**

17 studies (1206 patients) were included, and the overall quality of the included studies was moderate. In comparison with medical therapy, AMT has a better clinical efficacy for CIL (RR, 1.24; 95% CI, 1.17-1.32; *P* < 0.00001) and presents advantages in increasing leukocyte count (MD, 1.10; 95% CI, 0.67–1.53; *P* < 0.00001). Also, the statistical results show that AMT performs better in improving the CIL patients' Karnofsky performance score (MD, 5.92; 95% CI, 3.03–8.81; *P* < 0.00001).

**Conclusion:**

This systematic review and meta-analysis provides updated evidence that AMT is a safe and effective alternative for the patients who suffered from CIL.

## 1. Introduction

Nowadays, cancer imposes a major threat to human lives with high incidence and mortality. There were about 24.5 million cancer incident cases and 9.6 million deaths around the world in 2017 [1]. Along with radiation and surgery, chemotherapy remains one of the three standard therapeutic approaches against kinds of cancers. However, chemotherapy drugs are generally related to multiple side effects on patients, of which leukopenia (48.9%) is the most notable [2]. Leukopenia during cytotoxic chemotherapy is associated with an unfavorable prognosis in patients with breast cancer, colorectal cancer, and small-cell lung cancer [3]. Once chemotherapy-induced leukopenia occurs, it would not only hinders patients from completing prescribed treatment on time but also affects the quality of life (QoL), and even lead to serious infection and death. Currently, hematopoietic cytokine granulocyte, colony-stimulating factor (G-CSF), and granulocyte-macrophage colony-stimulating factor (GM-CSF) are widely employed to accelerate recovery for patients with CIL via accelerating neutrophil recovery and regulating granulopoiesis [4]. Although this adjuvant therapy is indeed effective with a rapid onset, the evidence of long-term efficacy and safety has not yet been determined. Besides, due to the high price, it exacerbates the cost of hospitalization and imposes additional economic burden for the families of cancer patients. Hence, effective and safe therapeutic strategies to reverse CIL without supporting tumor progression are urgently desired.

As an integral component of traditional Chinese medicine (TCM), acupuncture-moxibustion therapy is one of the most popular methods for complementing medicines in China and other East Asian countries. It is an external therapy to prevent and treat disease by stimulating particular acupoints on the human body with special needles or burning moxa [5]. In theory, acupuncture and moxibustion are two sides of the same coin, and in application, these treatments are supplementary to each other. Both acupuncture and moxibustion are based on the theory of channels and collaterals in Chinese medicine. Their effects are exerted by the stimulation of acupuncture points at specific anatomic locations on the body surface or tender points known as Ashi points, usually by needles or ignited moxa floss. The efficacy of AMT has been widely demonstrated in type 2 diabetes, dysenteric diarrhea, hepatogastrointestinal diseases, and many other diseases [6–9]. To investigate the therapeutic effect of AMT on CIL, many randomized clinical trials have been conducted in recent years. An exploratory meta-analysis [10] focused on chemotherapy-induced leukopenia published in 2007 demonstrated that acupoint stimulation promoted an increase in leukocyte count. In contrast, Chio et al. [11] argued that the evidence of the moxibustion efficacy in treating chemotherapy-induced leukopenia was insufficient to draw firm conclusions. To date, there continues to be no consensus about whether acupuncture-moxibustion therapy could be an attractive alternative for CIL. Therefore, this systematic review and meta-analysis was conducted to assess the efficacy and safety of AMT for the clinical treatment of CIL, so as to provide evidence for clinical practice.

## 2. Methods

This systematic review and meta‐analysis was performed in line with the Preferred Reporting Items for Systematic Reviews and Meta‐Analyses (PRISMA) schema and was registered at PROSPERO under registration number CRD42020152517 [12].

### 2.1. Literature Search Strategy

A comprehensive search was carried out in PubMed, Embase, Scopus, Web of Sciences, the Cochrane Library (CENTRAL), and four Chinese databases (Chinese Biomedical Database, Chongqing VIP Information, China National Knowledge Infrastructure, and WanFang Data) from their inception up to September 19, 2020 without language limitation. The search strategy consisted of Medical Subject Heading (Mesh) terms and free-text terms with logical operators. We performed an initial search of PubMed as follows: “Neoplasms” (Mesh) AND “Drug Therapy” (Mesh) AND “Leukopenia” (Mesh) AND “Acupuncture Therapy” (Mesh) AND “Moxibustion” (Mesh). The exploration of grey literature and hand-checking of key journals were completed to identify potentially relevant trials [13]. We also manually searched the references of the retrieved articles. EndNote X9 (Clarivate Analytics, 2018, Philadelphia, Pennsylvania) was utilized to store and manage all the literature downloaded from databases.

### 2.2. Study Selection

Studies were eligible in this meta-analysis, which met the following criteria. Study participant: the patient undergoing chemotherapy and diagnosed with leukopenia under clear criteria: (i) the total number of leukocytes remained below 4.0 or (ii) WHO grading standards. Study intervention: the patient in the experimental group was treated with acupuncture-moxibustion therapy. There were no restrictions on the selection of acupuncture points and the course of treatment. Comparison treatment: (i) conventional leukocyte-enhancing drugs (e.g., leucogen tablets, berbamine hydrochloride tablets, and rhG-CSF injection) or (ii) no comparator treatment. Study outcome: (i) primary measures: leukocyte count and total effective rate. Total effective rate = markedly effective + effective. Markedly effective: the count of leukocyte recovers to a normal value of 4.0 × 10^9^/L; effective: although the leukocyte count did not return normal, it increased (0.5∼1.0) × 10^9^/L; and invalid: no significant change in leukocyte count. (ii) Secondary measures: Karnofsky's index of performance status. Study design: randomized controlled trial. Additionally, the exclusion criteria included the following: (i) full-text not be obtained; (ii) incomplete data and the unclear outcome effect; (iii) duplicate publication; and (iv) case report, expert experience, review article, and other nonrandomized control trials.

After deleting the duplications, two reviewers (HMJ and YTZ) independently screened the title and abstract of studies according to the inclusion and exclusion criteria. Then, a full-text screening was performed by the same two reviewers. Any disagreement during study selection was resolved by consulting a third senior reviewer (YYX).

### 2.3. Data Collection and Quality Assessment

We designed a data extraction sheet to record related characteristics from the included articles, including diagnostic criteria, sample size, outcome measures, and treatment details. This was performed and proofread after completion by two authors (HMJ and YTZ) independently to ensure the accuracy of the extracted data. And then, they continued to assess the quality of included studies. The methodological quality was evaluated using the Cochrane risk of bias tool to assess the risk of bias in each included trial. The contents include six domains: random sequence generation, allocation concealment, blinding, integrity of outcome data, selective reporting, and other bias. Each item was rated three levels as low risk, high risk, and uncertain risk for insufficient information. Any disagreement in the assessment process was resolved through the third reviewer (YQF).

### 2.4. Statistical Analysis

All statistical analyses were performed using Review Manager software (version 5.3, Cochrane Collaboration, 2014, Copenhagen, Denmark). The risk ratios (RR) along with the corresponding 95% confidence intervals (95% CI) were calculated for dichotomous data, and mean differences (MD) with 95% CIs were calculated for continuous data. Statistical heterogeneity between studies was assessed by the *I*-square statistic (<25%, low heterogeneity; 25%–50%, moderate heterogeneity; and >50%, strong heterogeneity). A random-effects (RE) model was selected when heterogeneity existed significantly; otherwise, a fixed-effects (FE) model was preferred. In this meta-analysis, subgroup analysis and the sensitivity test were also conducted to explain the source of heterogeneity. Funnel plots were introduced to detect the existence of potential publication bias.

## 3. Results

### 3.1. Search Results

**Figure 1:**
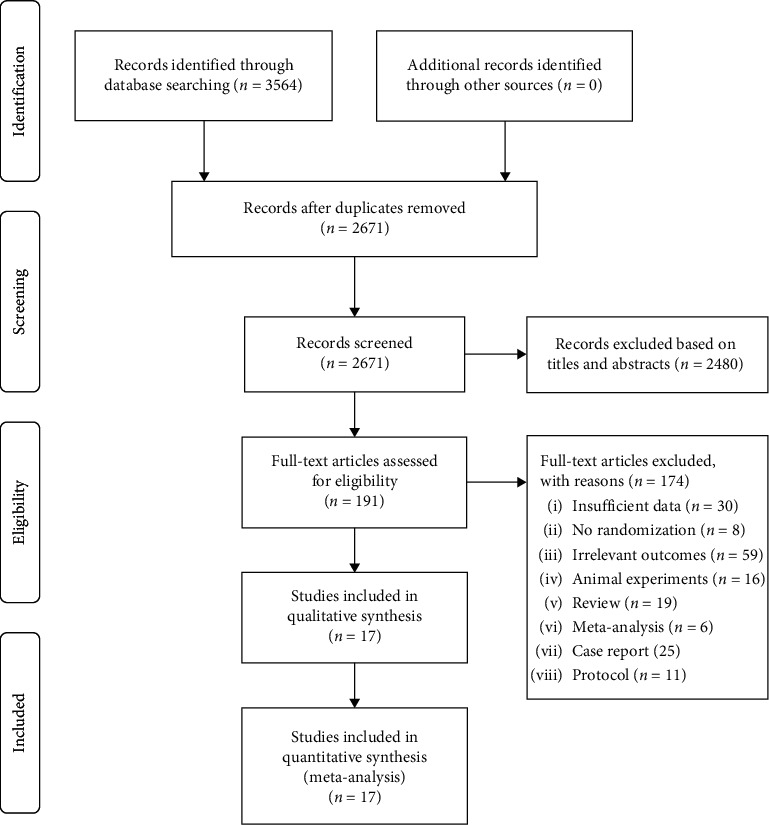
PRISMA flow diagram for literature search and study selection.

The database and manual searches yielded 3435 potentially relevant records, of which 849 were duplicates. Following two rounds of screening, 17 publications were identified in quantitative synthesis [14–30].  Figure 1 presents the process of study selection schematically based on PRISMA guidelines.

### 3.2. Study Characteristics

A total of 17 trials with 1206 participants were selected and varied widely in sample size (32–160 participants). All the included studies were conducted in China and published from 2010 to 2019. All patients fulfilled the diagnostic criteria of chemotherapy-induced leukopenia, with one study only confirmed in the title and abstract. Twelve studies adopted acupuncture therapy, while five studies relied on moxibustion therapy as the treatment of CIL. The primary outcome measures, i.e., the leukocyte count or clinical efficacy, could be obtained in all studies. The main characteristics and the specific interventions of each identified study are presented in Table 1.

### 3.3. Risk of Bias Assessment

**Figure 2:**
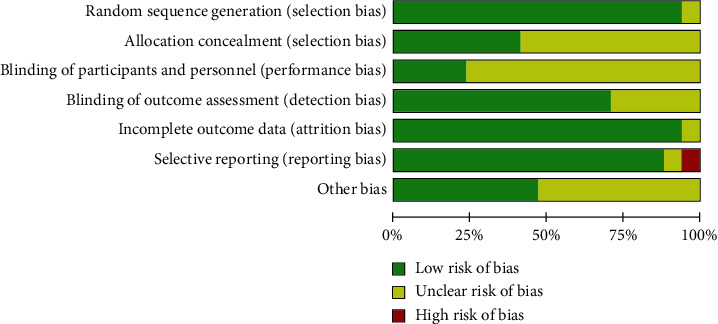
Risk of bias graph.

Figure 2 presents the quality assessment results of all eligible trials. The general quality of these trials was rated as moderate, since the relevant information was often missing or incomplete. All but one study [30] clearly mentioned the random allocation of participants to groups, in which 12 studies (70.1%) described the specific random methods [14, 16–18, 20, 22–24, 26–29]. Only one study adequately reported the allocation concealment, which was implemented using sealed opaque envelopes [16]. The risk of bias was uncertain for performance blinding of participants, personnel, and outcome assessors in most included studies. One study did not report the planned secondary outcomes of leukocyte count, so was ranked as high risk of reporting bias [19]. Most of studies (94.1%) were judged as low risk of bias from incomplete data, and no other potential sources of bias were detected.

### 3.4. Effects of Acupuncture-Moxibustion Therapy in Treating CIL

#### 3.4.1. Total Effective Rate

**Figure 3:**
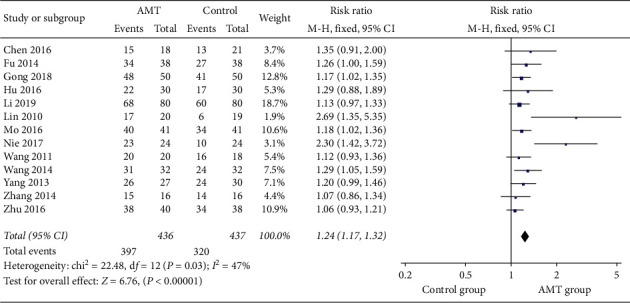
Forest plot of dichotomous data outcomes: total effective rate.

**Figure 4:**
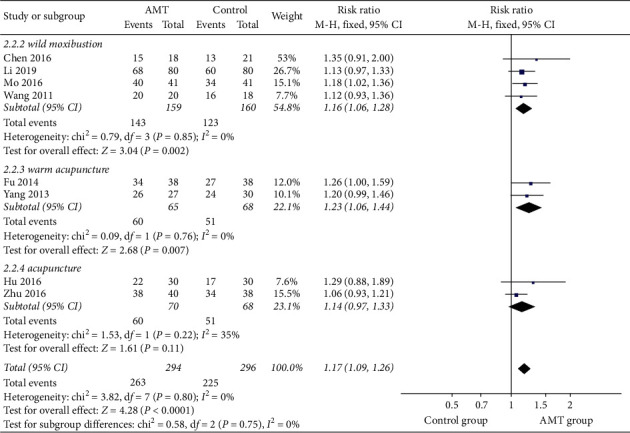
Subgroup analysis was performed based on different AMT types.

Thirteen studies involving 873 participants reported the total effective rate [14, 15, 18–21, 23–27, 29, 30]. Through the comparison with conventional treatment, AMT had a better clinical benefit in the treatment of leukopenia caused by chemotherapy (RR, 1.24; 95% CI, 1.17–1.32; *P* < 0.00001).  Figure 3 shows the combined results of 13 studies in this meta-analysis. Considering the clinical heterogeneity among these studies, we performed subgroup analysis based on the type of AMT to explore the source of heterogeneity [15, 18, 19, 23–26, 30]. As illustrated in Figure 4, the heterogeneity completely disappeared.

#### 3.4.2. Leukocyte Count

**Figure 5:**
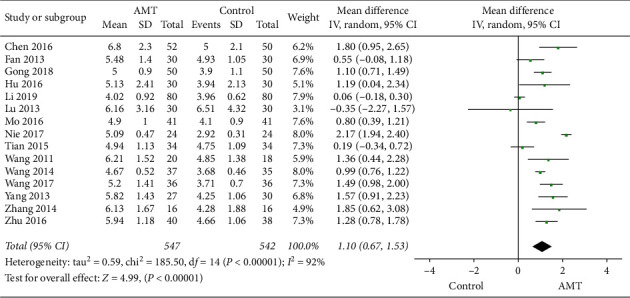
Forest plot of dichotomous data outcomes: leukocyte count.

**Figure 6:**
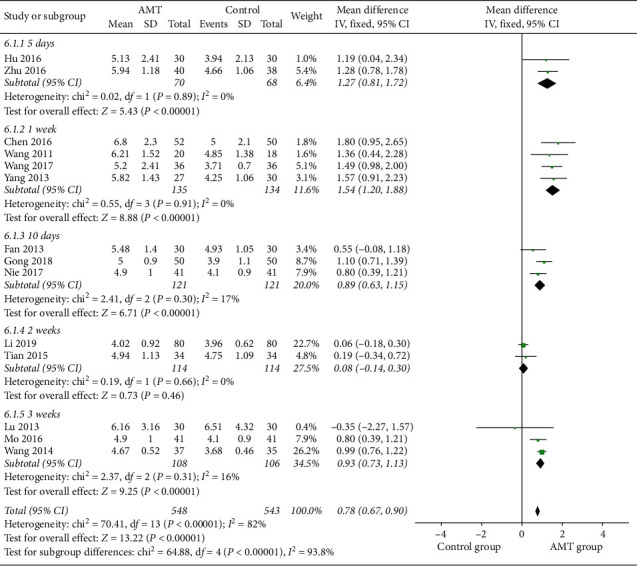
Subgroup analysis was performed based on the different duration of treatment.

The leukocyte count was recorded in 15 studies [15–18, 20–30] with 547 participants in experimental groups and 542 participants in control groups to estimate the therapeutic effect of AMT. Meta-analysis results indicated that acupuncture-moxibustion therapy was better than conventional medical therapy (MD, 1.10; 95% CI, 0.67-1.53; *P* < 0.00001), which is shown in Figure 5. Due to the significant heterogeneity, a subgroup analysis was carried out according to the duration of treatment to investigate the sources of heterogeneity [15–18, 20, 22–30]. The results of subgroup analyses partially explained the observed heterogeneity, which are displayed in Figure 6.

#### 3.4.3. Karnofsky Performance Score

**Figure 7:**
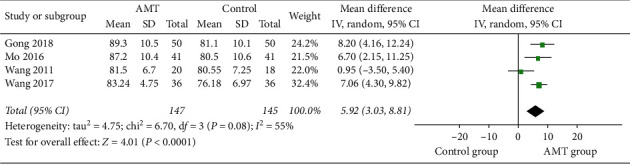
Forest plot comparing living quality between the AMT group and the control group.

Four studies [15, 26, 28, 29] with a total of 292 participants provided data on the Karnofsky performance score. The higher score represents the better functional health status. As shown in Figure 7, the results revealed that AMT performs better in improving health conditions (MD, 5.92; 95% CI, 3.03-8.81; *P* < 0.00001). Besides, we found that heterogeneity may originate from one study [15], and the heterogeneity disappeared after removing this single study.

### 3.5. Adverse Events

Safety outcome refers to the adverse events, such as allergic reaction, local responses (rash and scald), liver, and kidney injury, during the study. Among the conducted trials, safety outcomes were mentioned in five studies [15, 16, 18, 25, 28], four of which indicated that no adverse events had occurred. Explicitly, Fan [16] reported five (17%) participants in the control group and four (13%) in the experimental group had minor adverse events. The remaining 12 trials failed to assess safety for AMT treatment.

### 3.6. Sensitivity Analysis

To evaluate the robustness of pooled results, we performed the sensitivity analysis by omitting each study sequentially. Under the leave-one-out approach analysis, our results did not change noticeably.

### 3.7. Publication Bias

**Figure 8:**
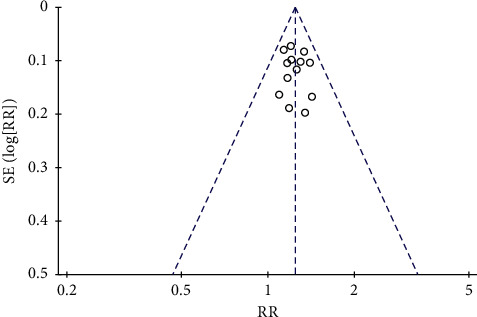
Funnel plot for assessing publication bias.

Publication bias was observed visually using funnel plot analyses. In this meta-analysis, the funnel plot for the total effective rate shaped with bilateral symmetry, indicating a lack of publication bias (Figure 8).

## 4. Discussion

### 4.1. Main Findings

As the meta-analysis reveals, acupuncture-moxibustion therapy could be an alternative treatment for chemotherapy-related leukopenia. From the clinical perspective, AMT could indeed increase the number of leukocyte and improve health status. Furthermore, there were almost no serious adverse events and treatment-related safety issues. We conducted subgroup analyses for each primary outcome, and the results suggested that different choices of AMT types and duration of treatment might be sources of heterogeneity. Based on our summaries about specific interventions of included studies, the most commonly used acupoints in treating CIL were Zusanli (ST36) and Guanyuan (CV4).

### 4.2. Possible Rationale of AMT for CIL

Chemotherapy-induced leukopenia is regarded as a potential serious adverse event, defined as white blood cell (WBC) count less than 4.0 × 10^9^/L during the course of chemotherapy. Patients with CIL typically present symptoms of loss of appetite, fatigue, insomnia, and dizziness. Based on the theory of traditional Chinese medicine (TCM), CIL belongs to the category of consumptive disease, owing to the exhaustion of genuine qi in the zang-fu viscera and the insufficiency of kidney essence and qi-blood. Researchers believe that there is an intimate association between the occurrence of malignant tumors and the deficiency of genuine qi. During attacking the cancer cells, chemotherapeutics also damaged the function of zang-fu viscera and qi-blood, leading to CIL. According to the theory of TCM and meridian, acupuncture-moxibustion is an ancient therapeutic modality that may be traced back more than 3500 years in China. Through meridian conduction, acupuncture-moxibustion therapy stimulates acupoints to strengthen the condition of zang-fu viscera and immune function, supporting genuine qi to improve symptoms of consumption. At the same time, some studies have attempted to further explore the molecular biological mechanism of acupuncture-moxibustion for relieving chemotherapy-induced leukopenia. By increasing the level of serum GM-CSF and G-CSF, acupuncture-moxibustion could prompt the proliferation and maturation of marrow hematopoietic cells, which favor the recovery of hematopoiesis function and reduce myelosuppression from chemotherapeutic agents [15]. Similarly, acupuncture-moxibustion upregulated the expression of cyclin D1 protein to accelerate the cell cycle progression, increasing DNA synthesis and enhancing DNA repair capacity. Therefore, leukocyte levels in the peripheral blood were elevated slowly but steadily under the influence of the protective effects on bone marrow cells by acupuncture-moxibustion stimulation [31].

### 4.3. Comparison of Previous Meta-Analysis

To the best of our knowledge, the present systematic review and meta-analysis is the first study on the effect and safety of AMT in treating CIL. Distinguished from the previous study [10, 11], we included twenty-one eligible trials with larger sample size, making the obtained conclusions more convincing than those of Lu et al. [10]. In addition, several different methods, including the laboratory indicator and clinical symptoms evaluation, were applied to estimate the therapeutic effect of AMT. From various aspects, this embodies a significant role of AMT in improving the quality of life and alleviating chemotherapy side effects. Also, it is worth pointing out that we take adverse events related to AMT into account, which were previously ignored by Choi et al. [11].

### 4.4. Limitations

First, even though comprehensive literature search was performed, we only identified studies published in Chinese. So, the potential language bias might be caused. Second, there were no uniform standards for the selection of acupoints of patients with leukopenia after chemotherapy. By combining experience in clinical practice, clinicians might have preferences for different acupoints regimens. Finally, the methodological quality of included trials was deemed weak to moderate. Hence, interpretations of our findings should be taken with caution.

With the above limitations, more multicenter randomized clinical trials with rigorous design should be conducted to clarify the efficiency and safety of acupuncture-moxibustion therapy in CIL. Meanwhile, the most optimal regimen of acupoint selection for CIL treatment needs to be investigated in future research. Besides, further studies with extended follow-up periods are required to determine the long-term efficacy and benefits of AMT in CIL.

## 5. Conclusion

In summary, this is an updated systematic review and meta-analysis, revealing that the AMT, as a nonpharmaceutical intervention, could be an effective and safe alternative for patients undergoing CIL. Despite limitations, the study provides the direction and basis for future research. High-quality randomized controlled trials are warranted to further validate current results, offering more confident conclusions for clinical practices.

## Figures and Tables

**Table 1:** Characteristics of included studies.

No.	Study ID	Diagnostic criteria	Number (T/C)	Age, years (T/C)	Interventions				Outcomes
					Control group	Treatment group	Duration	Adverse events	
1	Lin et al. [14]	The total number of leukocytes remained below 4.0	20/19	52.1 ± 7.65/53.7 ± 6.14	Oral medicine (leucogen tablets)	Acupoint injection at ST36 with Shenmai injection	1× per day for 21 days (21 total)	Not mentioned	TER

2	Wang [15]	The total number of leukocytes remained below 4.0	20/18	55.35 ± 9.73/54.80 ± 8.15	Oral medicine (blood increasing pill)	Moxibustion at ST36, CV4, and CV6	1× per day for 7 days (7 total)	No local scalds and no allergies or discomfort caused by other treatments during and after treatment	LC, TER, and KI

3	Fan [16]	The total number of leukocytes remained below 4.0	30/30	57.72 ± 12.09/61.59 ± 12.78	Intravenous drip of Shenqi Fuzheng injection	Ginger moxibustion at GV14, BL20, BL23, BL17, CV12, CV14, and CV4 for 4 moxa cone	1× per day for 10 days (10 total)	A total of 4 cases of adverse events occurred (two were intolerable to moxa smoke, one had blisters from moxibustion, and one treated middle back pruritus).	LC

4	Lu et al. [17]	WHO grading standards	30/30	30–76/40–75	Intravenous drip of granisetron hydrochloride injection	Intravenous drip of granisetron hydrochloride injection + acupoint injection at ST36 with Aidi injection	ND	Not mentioned	LC

5	Yang [18]	The total number of leukocytes remained below 4.0	27/30	56.04 ± 10.98/54.10 ± 11.02	rhG-CSF injection	rhG-CSF injection + moxibustion with warming needle at ST36, BL17, CV4, and CV6	1× per day for 7 days (7 total)	No signs of infection	LC and TER

6	Fu et al. [19]	The total number of leukocytes remained below 4.0	38/38	55–79; mean: 61.5 ± 8.9	Oral medicine (leucogen tablets and batilol tablets)	Moxibustion with warming needle at ST36, SP6, SP9, SP10, GV14, BL20, PC6, CV4, and CV6	1× per day for 14 days (14 total)	Not mentioned	TER

7	Wang et al. [20]	WHO grading standards	37/35	54.24 ± 7.89/51.25 ± 8.97	Oral medicine (leucogen tablets and batilol tablets)	Moxibustion at ST36, SP6, SP10, CV4, and CV8 for 4 moxa cone	(1× per day for 14 days, rest 7 days) ^*∗*^2 (28 total)	Not mentioned	LC and TER

8	Zhang [21]	The total number of leukocytes remained below 4.0	16/16	60.06 ± 7.54/61.38 ± 8.40	rhG-CSF injection	Moxibustion with seed-sized moxa cone at ST36 and GV14 for 9 moxa cone	1× per day	ND	LC and TER

9	Tian [22]	WHO grading standards	34/34	32–76; mean: 51.6 ± 4.3	Oral medicine (leucogen tablets and batilol tablets)	Moxibustion at ST36, CV4 and CV6 for 15 minutes	1× per day for 14 days (14 total)	Not mentioned	LC

10	Hu [23]	The total number of leukocytes remained below 4.0	30/30	1.30 ± 9.64/52.03 ± 8.87	Oral medicine (batilol tablets) or subcutaneous injection of rhG-CSF injection	Oral medicine (batilol tablets) or subcutaneous injection of rhG-CSF injection + acupuncture at ST36, SP6, SP10 and BL23 for 30 minutes	1× per day for 5 days (5 total)	Not mentioned	LC and TER

11	Zhu [24]	The total number of leukocytes remained below 4.0	40/38	47 ± 16/46 ± 14	Oral medicine (batilol tablets) or subcutaneous injection of rhG-CSF injection	Oral medicine (batilol tablets) or subcutaneous injection of rhG-CSF injection + acupuncture at ST36, SP6, BL18, BL23, CV4, and SP10	1× per day for 5 days (5 total)	ND	LC and TER

12	Chen and Xu [25]	Definitive diagnosis without basis	52/50	38–72/37–70	Oral medicine (leucogen tablets and berbamine hydrochloride tablets)	Oral medicine (leucogen tablets and berbamine hydrochloride tablets) + moxibustion at ST36 for 15 minutes	1× per day for 7 days (7 total)	No adverse events found	LC and TER

13	Mo et al. [26]	WHO grading standards	41/41	55 ± 10/56 ± 10	Oral medicine (leucogen tablets and batilol tablets)	Moxibustion at ST36, CV4, CV6, and CV8 for 20 minutes	6× per week for 3 weeks (18 total)	Not mentioned	LC, TER, and KI

14	Nie [27]	The total number of leukocytes remained below 4.0	24/24	49.09 ± 8.01/51.2 ± 6.04	rhGM-CSF Injection	Moxibustion at GV14, CV4, CV8, BL17, BL20, and BL23	1× per day for 10 days (10 total)	Not mentioned	LC and TER

15	Wang [28]	The total number of leukocytes remained below 4.0	36/36	57.21 ± 7.37/57.30 ± 8.37	Oral medicine (Shenqi tablets)	Acupoint injection at ST36 with Shenfu injection	1× per day for 7 days (7 total)	No obvious adverse events such as skin rashes and allergies during the study	LC and KI

16	Gong et al. [29]	WHO grading standards	50/50	4.17 ± 5.71/51.49 ± 6.27	Oral medicine (batilol tablets and vitamin B4 tablets)	Ginger moxibustion at CV4, CV6, and CV8	1× per day for 10 days (10 total)	Not mentioned	LC, TER, and KI

17	Li et al. [30]	The total number of leukocytes remained below 4.0	80/80	8.35 ± 9.27/66.70 ± 12.13	Subcutaneous injection of rhG-CSF injection	Subcutaneous injection of rhG-CSF injection + moxibustion at ST36, CV4, CV6, BL23, and GV4	1× per day for 14 days (14 total)	Not mentioned	LC

WHO, World Health Organization; T, treatment group; C, control group; rhG-CSF, recombinant human granulocyte colony-stimulating factor injection; rhGM-CSF, recombinant human granulocyte/macrophage colony-stimulating factor injection; ND, not determined; LC, leukocyte count; TER, total effective rate; KI, Karnofsky's index.

## Data Availability

All articles retained for this review were made available to the public through PubMed, Embase, the Cochrane Library, Scopus, Web of Sciences, Chinese BioMedical Database, Chongqing VIP Information, China National Knowledge Infrastructure, and WanFang Data. All data analysed in this study are included in the published articles.
